# Supplementary material for: NMJ-Analyser identifies subtle early changes in mouse models of neuromuscular disease
Source: Sci Rep. 2021 Jun 10;11:12251. doi: 10.1038/s41598-021-91094-6 (PMC8192785; doi:10.1038/s41598-021-91094-6)
Supplement: Supplementary file 1 — Supplementary Informations. [file 41598_2021_91094_MOESM1_ESM.docx]

**NMJ-Analyser identifies subtle early changes in mouse models of neuromuscular disease**

Alan Mejia Maza^1^, Seth Jarvis^1^, Weaverly Colleen Lee^1^, Thomas J. Cunningham^2^, Giampietro Schiavo^1,3^, Maria Secrier^4^, Pietro Fratta^1^, James N. Sleigh^1,3^, Carole H. Sudre^5,6,7^**^, ❋,✢^** & Elizabeth M.C. Fisher^1,^ **^✢^**

^1^  Department of Neuromuscular Diseases, UCL Queen Square Institute of Neurology, University College London, London WC1N 3BG, UK.

^2^  Mammalian Genetics Unit, MRC Harwell Institute, Oxfordshire, OX11 0RD, UK.

^3^ UK Dementia Research Institute, University College London, London WC1E 6BT, UK.

^4^  Department of Genetics, Evolution and Environment, UCL Genetic Institute, University College London, London WC1E 6BT, UK.

^5^ MRC Unit for Lifelong Health and Ageing, Department of Population Science and Experimental Medicine, University College London, London WC1E 6BT, UK.

^6^  Centre for Medical Image Computing, Department of Computer Science, University College London, London WC1E 6BT, UK.

^7^ School of Biomedical Engineering and Imaging Sciences, King's College London, London W2CR 2LS, UK.

**^❋^** Corresponding author. E-mail: c.sudre@ucl.ac.uk

^✢^ These authors contributed equally

**Additional File 1**

**
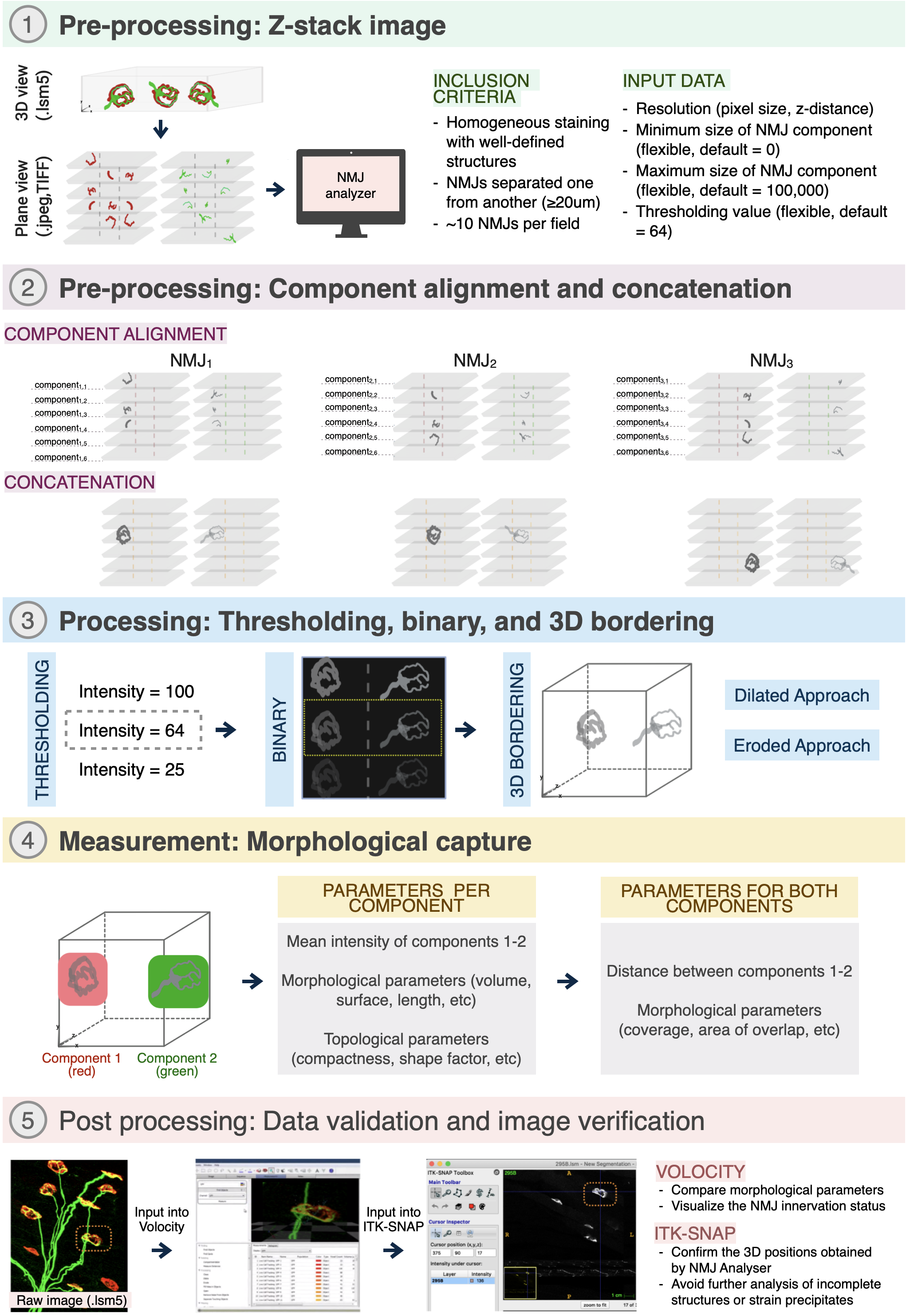
**

**Fig.S1 Workflow of image morphological analysis using NMJ-Analyser.** Morphological analysis of NMJs requires (1) pre-processing, (2) processing, (3) measurement and (4) post-processing of images. (1) Images are required to be in adequate format for identification of NMJ innervation status and for posterior capture of morphological features (3D and plane images, respectively). (2) Pre-processing step is used for capturing automatic morphological features. Images concatenation is used to assigned a 3D position to each NMJ component (centre of mass, [*P* (𝚡, 𝚢, 𝚣)]). (3) Processing refers to the cut-off intensity used to define and differentiate the staining from background (thresholding). It is important to keep the same threshold between batches for reproducibility. (4) Measurement refers to the capture of morphological features of pre- and post-synaptic NMJ. (5) Post-processing or manual curation is required as NMJ-Analyser gives a large output automatically. When analysing images in a high throughput manner, antibody precipitates or incomplete NMJs may be identified and their features collected. Thus, using the 3D position of individual objects identified in step 2-4 can be clearly identified using ITK-SNAP viewer (freely available). These antibody precipitates or incomplete NMJs identified can be then easily dropped for further analysis.

**
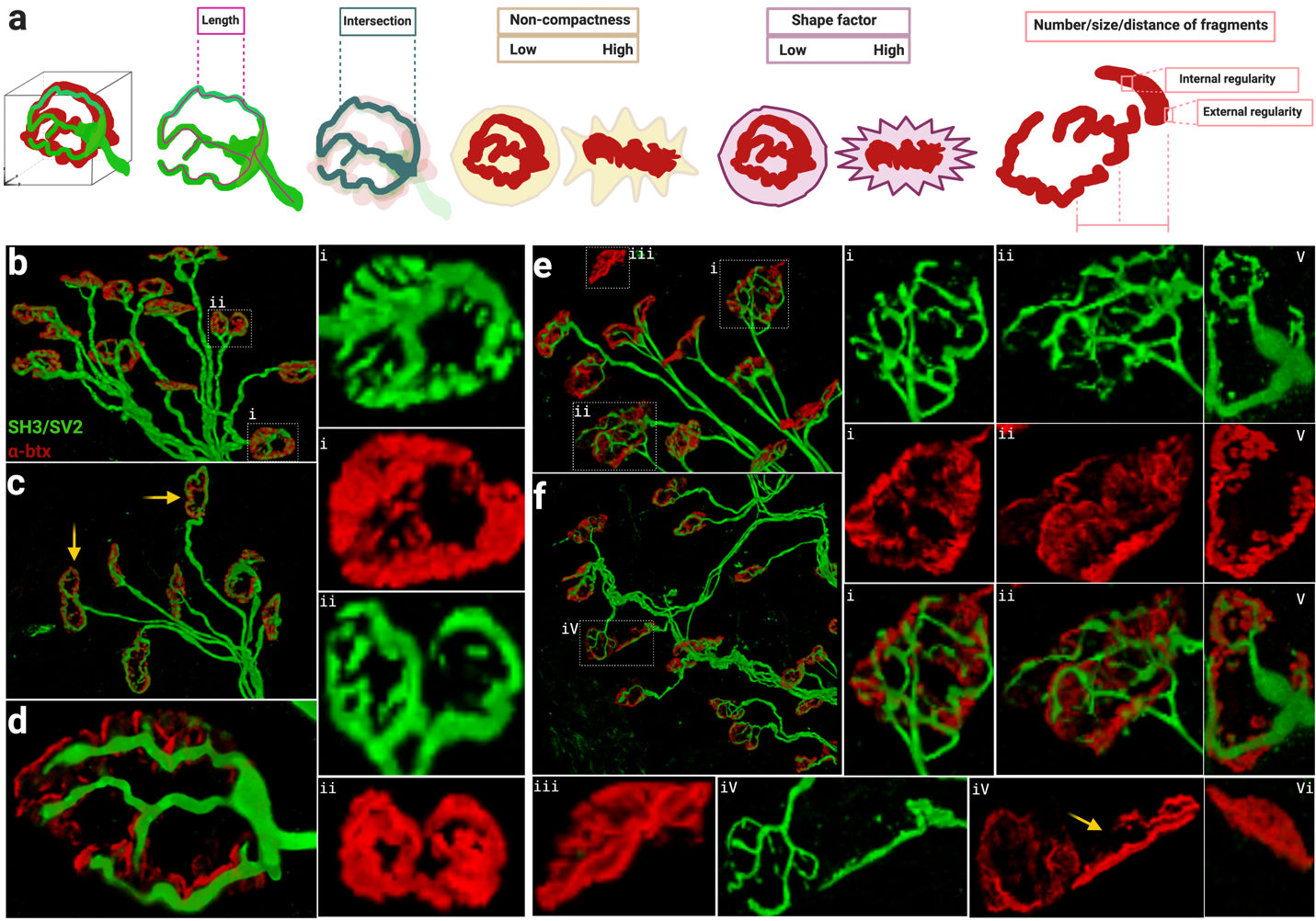
**

**Fig. S2. Morphological characteristics of wildtype and degenerating NMJs**. a) schematic representation of some features detected by NMJ-Analyser. b-f) Confocal images of healthy and degenerating NMJs visualized using nerve terminal markers (2H3+SV2, green) and motor endplate staining (alpha bungarotoxin, α-btx, red). b-d figures and their insets are wildtype 1-month old NMJs. NMJs look fully formed and innervating axon covers most of the motor endplate (i, ii and d, yellow arrows). Based on the shape of WT pre- and post-synaptic structures (b-i, b-ii; insets), the non-compactness and shape factor values should be lower than more amorphous elements such as mutant NMJ components observed in e and f figures (e-iii and f-iv; insets). e-f and insets v-vi correspond to Gars synapses of the lumbrical muscle. Disorganization of pre-synaptic axon is evident (i,ii, iv and v) in association (iv,v, yellow arrow) or not (i) with evident motor endplate alteration. Insets iii and vi display vacant motor endplates.

**
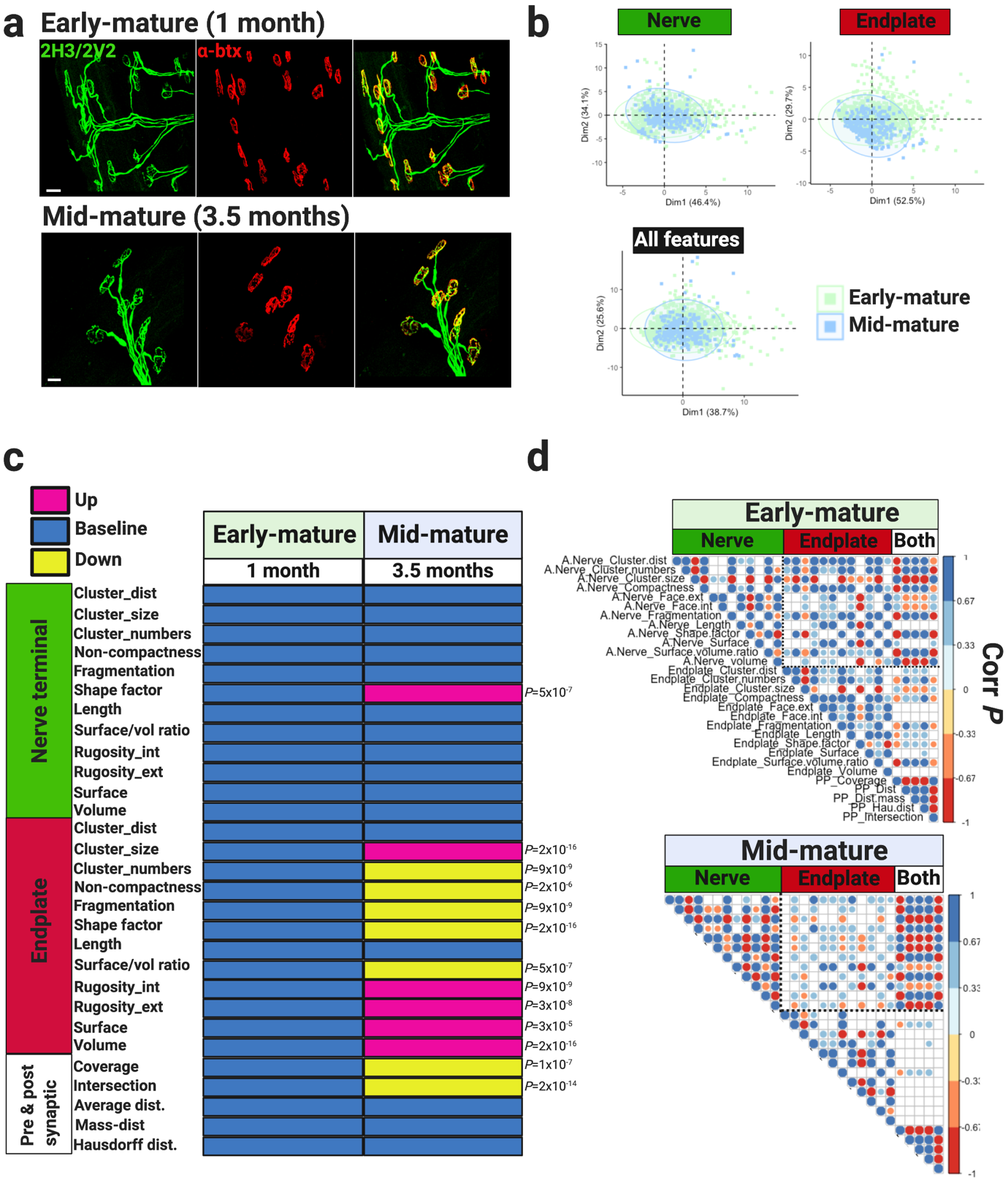
**

**Fig.S3 Structural features of healthy NMJs in male C57BL/6J-SJL mice.** (a) Confocal images of NMJs visualized using nerve terminal markers (2H3+SV2, green) and motor endplate staining (α-btx, red). Early- and mid-mature NMJs display mono-innervated axonal input and pretzel-like shape. (b) PCA plot showing no major cluster differentiation between early- and mid-mature NMJs. (c) Module displaying NMJ morphological parameters measured in early- and mid-mature NMJs. Early-mature NMJs are considered as baseline values. Morphological parameters were analysed using a using Wilcoxon signed-rank test (d) Matrix correlation plots of nerve terminal and motor endplate parameters, and interaction between them measured in early- and mid-mature NMJs. Positive and negative correlations are given by blue and red scales, respectively (*P*-value ≤ 5x10^-2^). Datapoint showed non-normal distribution and spearman correlation tests were used. Empty circles indicate no significant *P* -value was observed. Number of mice, early- and mid-mature: (5+4). Scale bars = 20μm.

**
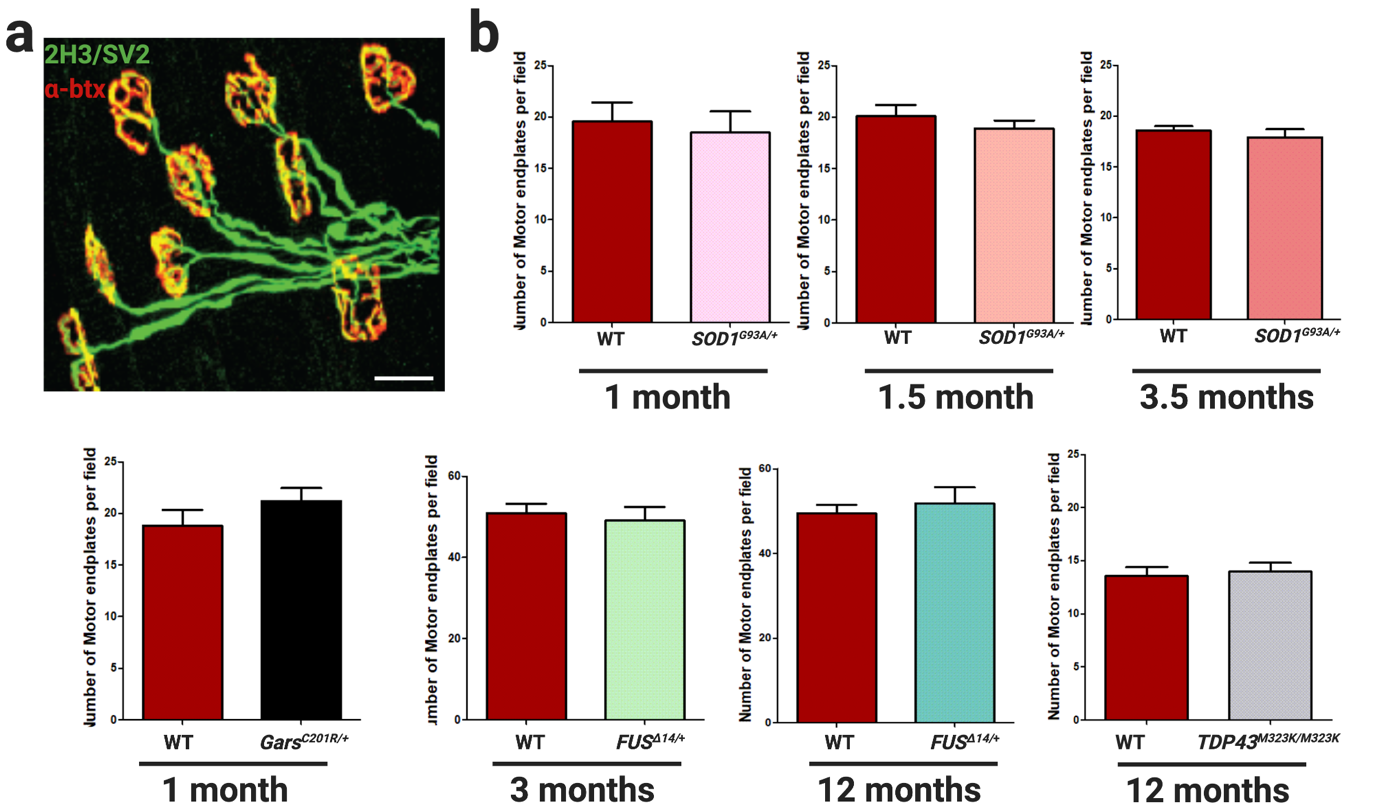
**

**Fig.S4 Motor endplate counts in ALS and CMT2D mouse models.** (a) Lumbrical NMJs from 1.5-month-old WT mice visualized using nerve terminal markers (2H3+SV2, green) and motor endplate staining (α-btx, red). (b) Bars represent average of motor endplates per field; 8-12 fields per mouse were counted. Number of mice, WT and mutant: *SOD1^G93A/+^*, 1-month (5+5), 1.5-month (5+5), 3.5-months (4+9); *Gars^C201R/+^* (5+5); *FUS^Δ14/+^*, 3-months (4+4), 12-months (4+4); *TDP43^M323K/M323K^*, 12-months (5+5). Mean ± S.E.M were plotted. Scale bar = 20μm.

**
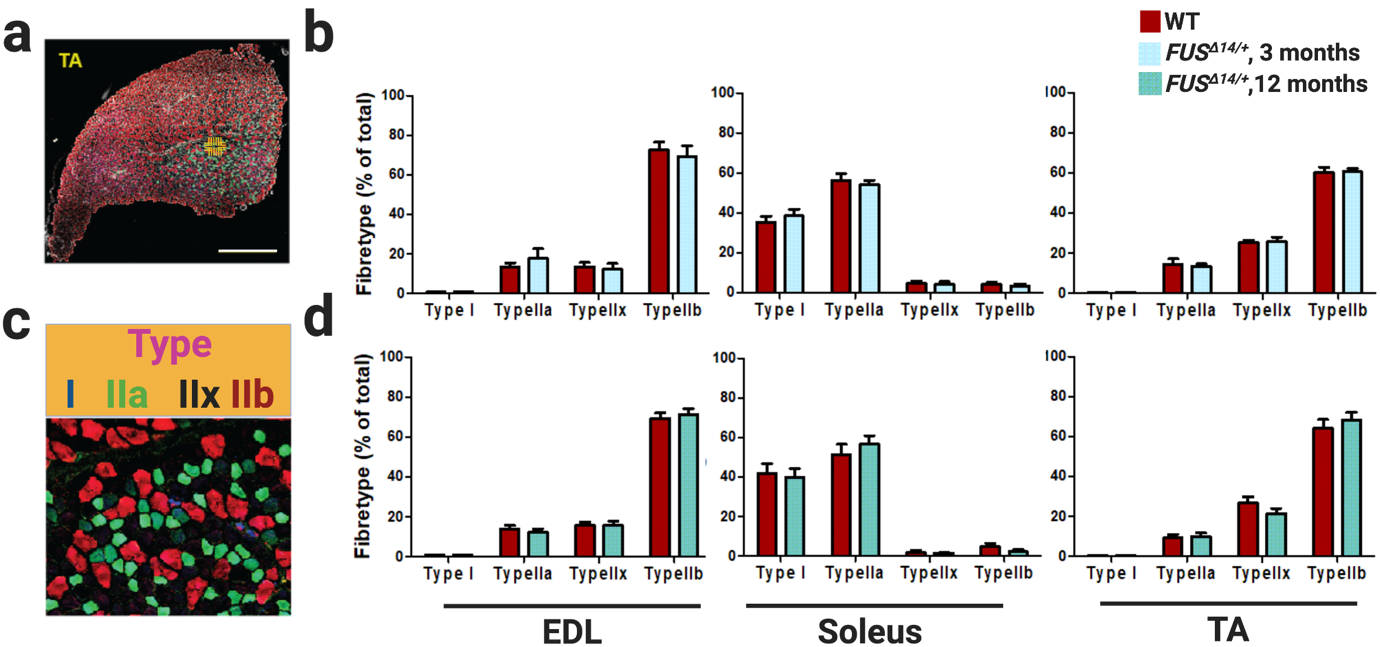
**

**Fig.S5 Fibretype composition in hindlimb muscles of *FUS^Δ14/+^* mice.** (a) Transversal section of TA muscle visualized using fibre markers type I, IIa and IIb. (b) Fibretype composition of EDL, soleus and TA hindlimb muscles in 3-month old WT and FUS^Δ14/+^ littermates. (c) High-magnification of TA shown in (a), displaying individual fibretypes. Fibretype I (blue), IIa (green), IIx (negative staining) and IIb (red). (d) Fibretype composition of EDL, soleus and TA hindlimb muscles in 12-month old WT and *FUS^Δ14/+^* littermates. Number of mice, WT and *FUS^Δ14/+^*: 3-months (7+8), 12-months (7+8). Male and female mice at each timepoint (3+4 or 4+4). No significant sex-differences were observed. Mean ± S.E.M were plotted. Scale bar = 20μm.

**
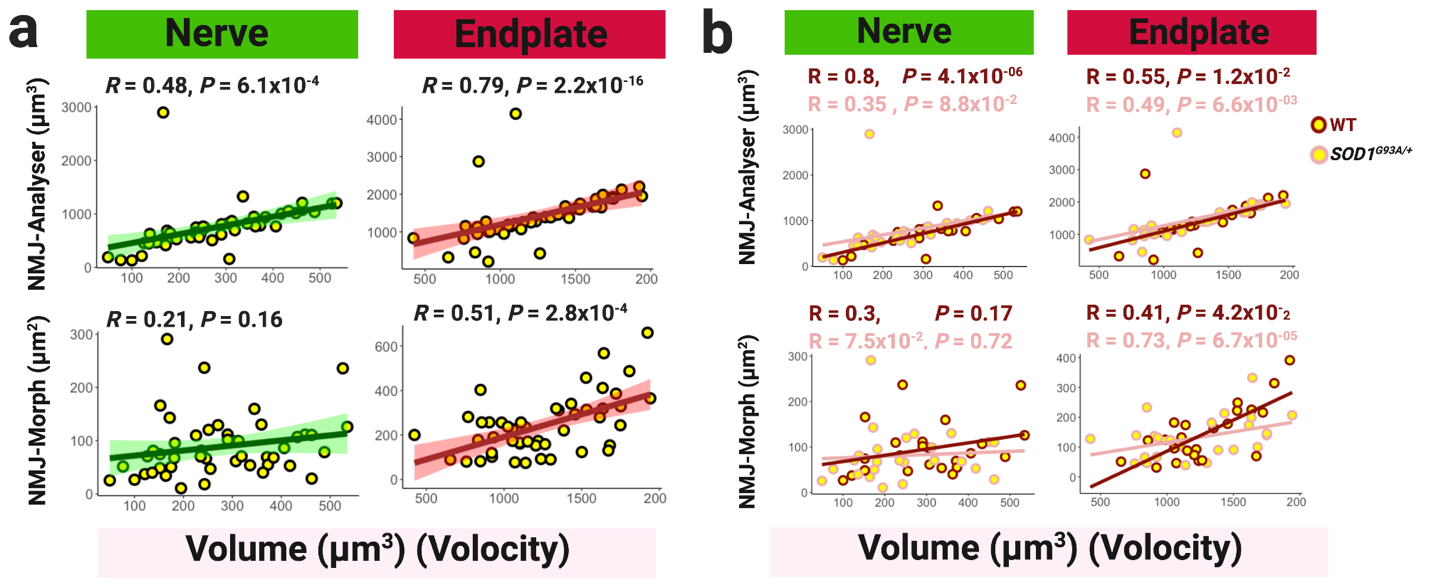
**

**Fig.S6 Performance of NMJ-Analyser and NMJ-Morph using batch 2.** (a) Correlation of nerve terminal and endplate output obtained using NMJ-Analyser and NMJ-Morph. Datapoint showed non-normal distribution and spearman correlation tests were used. Green and red shading represent the confidence interval. (c) Correlation of nerve terminal and endplate output obtained using NMJ-Analyser and NMJ-Morph, divided by genotype.

**Table S1 Total number of fibres in hindlimb muscles**

|  | **3-months** | | **12-months** | |
| --- | --- | --- | --- | --- |
|  | **WT** | ***FUS^Δ14/+^*** | **WT** | ***FUS^Δ14/+^*** |
| **Soleus** | 861 ± 37 | 789 ± 66 | 841 ± 59 | 832 ± 57 |
| **EDL** | 765 ± 87 | 837 ± 75 | 877 ± 58 | 869 ± 62 |
| **TA** | 2470 ± 208 | 2654 ± 172 | 2605 ± 241 | 2412 ± 250 |

**Table S2 Total number of NMJs analysed**

|  |  | ***SOD1 ^G93A/+^* (n=2078)** | ***Gars^C201R/+^***  **(n=1343)** | ***FUS^Δ14/+^* (n=403)** | ***TDP43^M323K/M323k^***  **(n=95)** | **WT**  **(2809)** |
| --- | --- | --- | --- | --- | --- | --- |
| **Innervation**  **status** | **Fully** | **1664** | **340** | **354** | **93** | **2713** |
|  | **Partial** | **77** | **222** | **24** | **2** | **63** |
|  | **Denervated** | **337** | **782** | **25** | **-** | **33** |
| **Maturity** | **Early** | **1422** | **408** | **-** | **-** | **1929** |
|  | **Mid** | **656** | **935** | **139** | **-** | **440** |
|  | **Late** | **-** | **-** | **264** | **95** | **440** |
| **Muscle** | **Lumbricals** | ***2078*** | **408** | **403** | **95** | **2809** |
|  | **FDB** | **-** | **935** | **-** | **-** | **-** |
| **Phenotype** | **Heathy** | **1664** | **340** | **354** | **93** | **2713** |
|  | **Degenerating** | **414** | **1003** | **50** | **2** | **96** |

**Additional File 2**

**NMJ-Analyser Tutorial**

**Step 1: Installation of Python**

- Python3 (or older version) are required for proper functioning of NMJ-Analyser
- Installing “Miniconda” for windows 10 users.

**Step 2: Installation of modules**

- pip3 (for Mac OSX) or pip (for Windows), scipy, pillow, glob, os-sys, numpy and argparse modules need to be installed on your machine before working with NMJ-Analyser.

**Step 3: NMJ_Analyser installation**

- Using GUI interface

**Step 4: Running NMJ_Analyser**

**Step 1.- Installation of Python**

- Python3 is required for NMJ-Analyser installation. If python3 is not installed, NMJ-Analyser will not work.

**Mac OSX:**

- On your Mac, type ‘terminal’ in spotlight search. A command line window pops up and type “python3 –version”. As a result, you should get “Python V3.x.x.” as an output (or the installed python version on your machine). If error pops up, you need to install python3 on your Mac OSX.
- To install python3 in your machine, please go to the following link: <https://www.python.org/downloads/mac-osx/>

**Windows:**

- On your Windows machine: type “cmd” in Start menu. A command line window pops up and type “python —version” (python*hypen*hypen*version) or “python -V” to confirm it is installed. As a result, you should get “Python 3.x.x.” as an output (or the installed python version on your machine).. If error pops up, you need to install python3 on your machine.
- For simplicity, we use “Miniconda" environment to install python modules when working in windows. Please download Miniconda “32” or “64” depending of your machine <https://docs.conda.io/en/latest/miniconda.html>. This environment comes with Python 3.8 pre-installed by default.
- To confirm python is successfully installed in your machine, please type “miniconda” in the Start menu. A Miniconda command line should come up (similar to the “cmd” command).
- In the miniconda command line type “python -- version” (python*hypen*hypen*version). As a result, you should get “Python 3.x.x.” as an output.

**Step 2.- Installation of modules**

Please install the following modules installation:

- sciPy, for multidimensional image processing: <https://www.scipy.org/install.html>
- pillow, for reading images: https://pillow.readthedocs.io/en/stable/installation.html
- glob, for finding the pathnames: <https://docs.python.org/3/library/glob.html>
- os-sys: https://pypi.org/project/os-sys/
- numpy: <https://numpy.org/install/>
- argparse: <https://pypi.org/project/argparse/>
- pandas: https://pandas.pydata.org/pandas-docs/stable/getting_started/install.html
- sys: <https://docs.python.org/3/install/>
- Scikit-image:<https://scikit-image.org/docs/dev/install.html#install-via-pip>

**Mac OSX:**

- pip3 installation is critical for NMJ-Analyser. If Python3 or older version is installed, pip3 comes installed by default. To confirm pip3 is pre-installed type “pip3 --version” in the command line of your Mac OSX.
- If pip3 is not preinstalled by default. On the command line type “sudo easy_install pip” to install pip3 (please, notice the underscore between ´easy and install´). You will be required to type your administrative password.


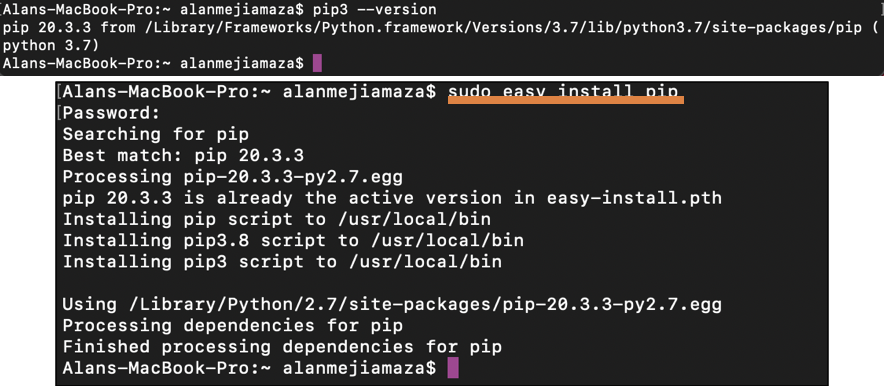


To install modules on your Mac OS X terminal type:

- pip3 install scipy
- pip3 install pillow
- pip3 install glob2
- pip3 install numpy
- pip3 install pandas
- pip3 install nibabel
- pip3 install scikit-image (or python -m pip install -U scikit-image) or pip3 install skimage
- pip3 install tk

Most of these packages should be installed by default. Please verified that all packages were installed correctly.

**Windows 10:**

On the miniconda terminal, type:

- conda install pip
- conda install scipy ( or “conda install -c anaconda scipy”)
- conda install pillow
- conda install pandas
- conda install numpy
- conda install glob2
- conda install scikit-image
- conda install -c conda-forge nilabel (or conda install nibabel)
- conda install tk
- conda install wheel
- Please notice that depending on the python/conda version some commands may change a

bit. If the “conda install ‘package_name’ “ won’t work, try replacing ‘conda by pip’: “ pip install ‘package_name’ “ in the same miniconda terminal.

- Please proceed to update previous installed packages if necessary when installing


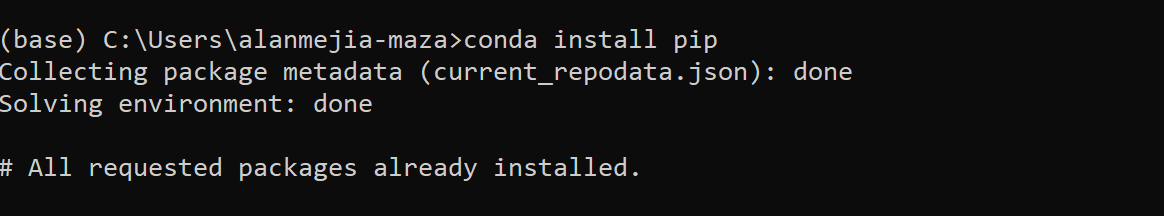


**Step 3.- NMJ-Analyser installation**

**Mac OSX**

- At this stage, Python3 or older version and modules should be correctly installed. Please refer to the following link if have any troubles. https://pip.pypa.io/en/stable/installing/
- Open the terminal and copy “pip3 install nmjanalyzer”. It will automatically start the installation. Please write exactly as it is to avoid errors. If errors persist, type in the command line “pip install -i https://test.pypi.org/simple/ nmjanalyzer”. The software is hosted at <https://pypi.org/project/nmjanalyzer/>


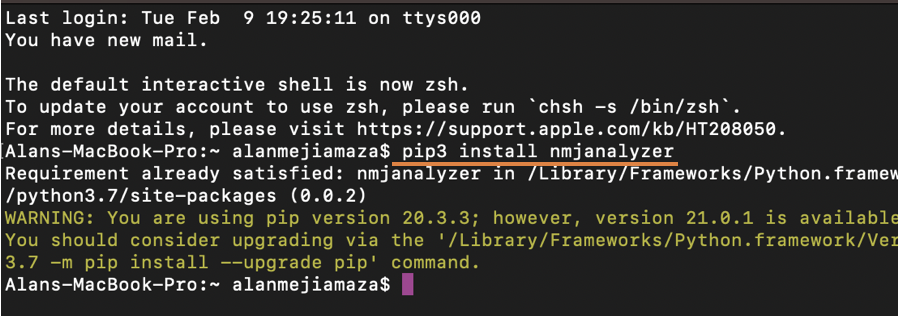


-
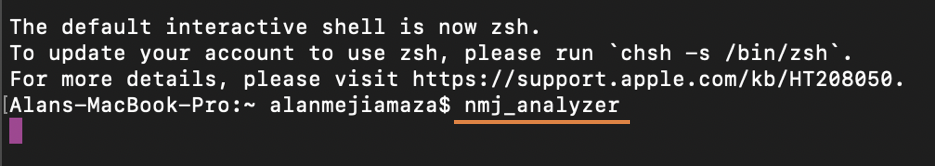
To start NMJ-Analyser interface, type “nmj_analyzer” in the terminal (nmj*underscore*analyzer)
- If GUI interface (see below) come up automatically, NMJ-Analyser is successfully installed.


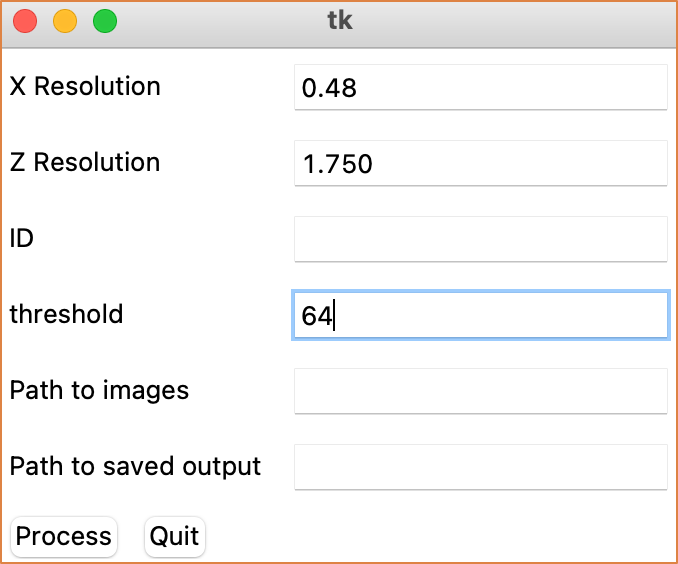


**Windows**

- After installation of conda, python and modules using the Miniconda terminal, type “pip install nmjanalyzer” in the miniconda terminal.
- Then, type “nmj_analyzer” (or NMJ_ANALYZER) in the command line of the miniconda terminal. Depending on your computer properties, this make take few seconds or minutes.


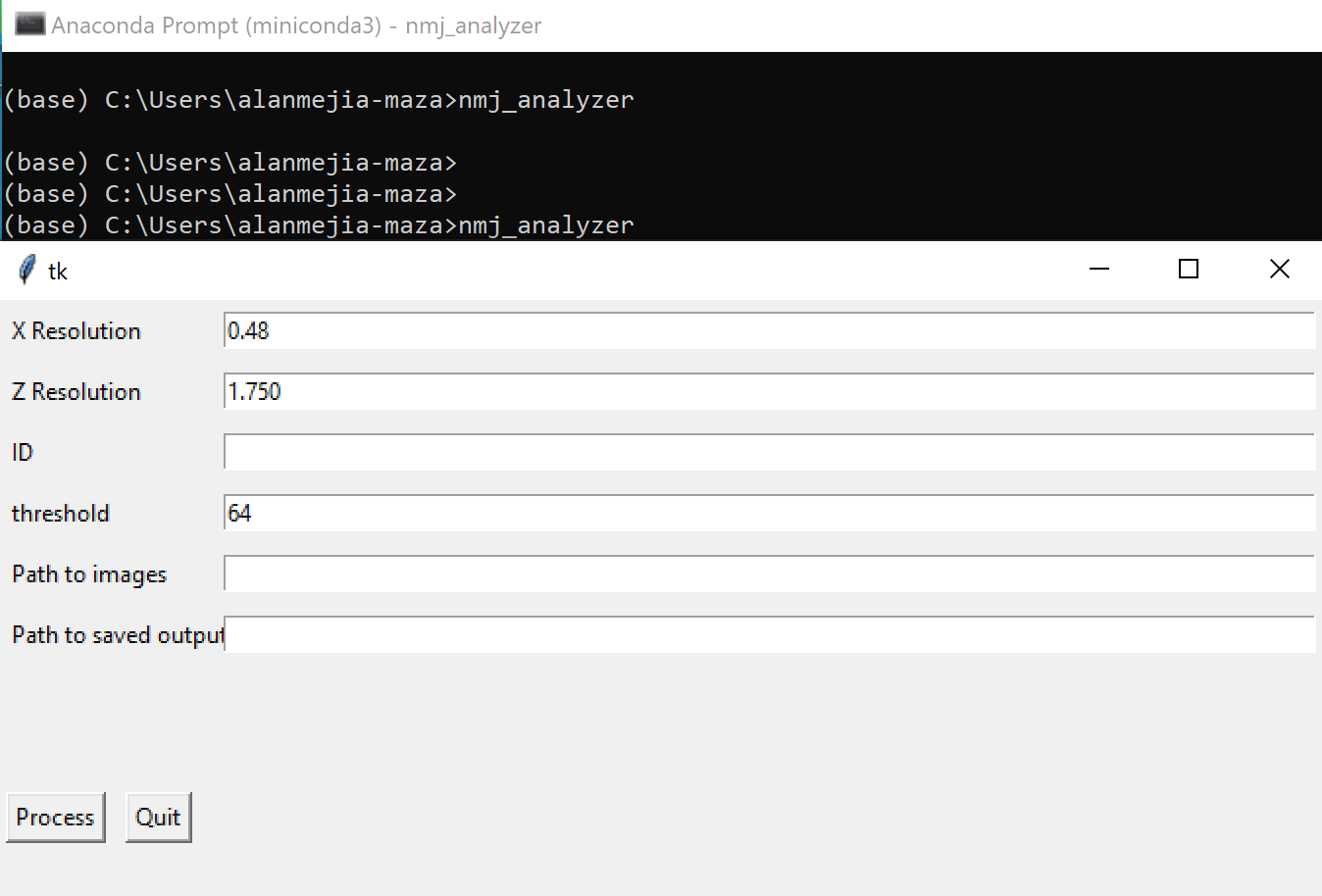


**Step 4.- Running NMJ_Analyzer**

- Download the MouseTest_sample.zip file from <https://github.com/csudre/NMJ_Analyser> (See below, click in “Code -> Download ZIP”)


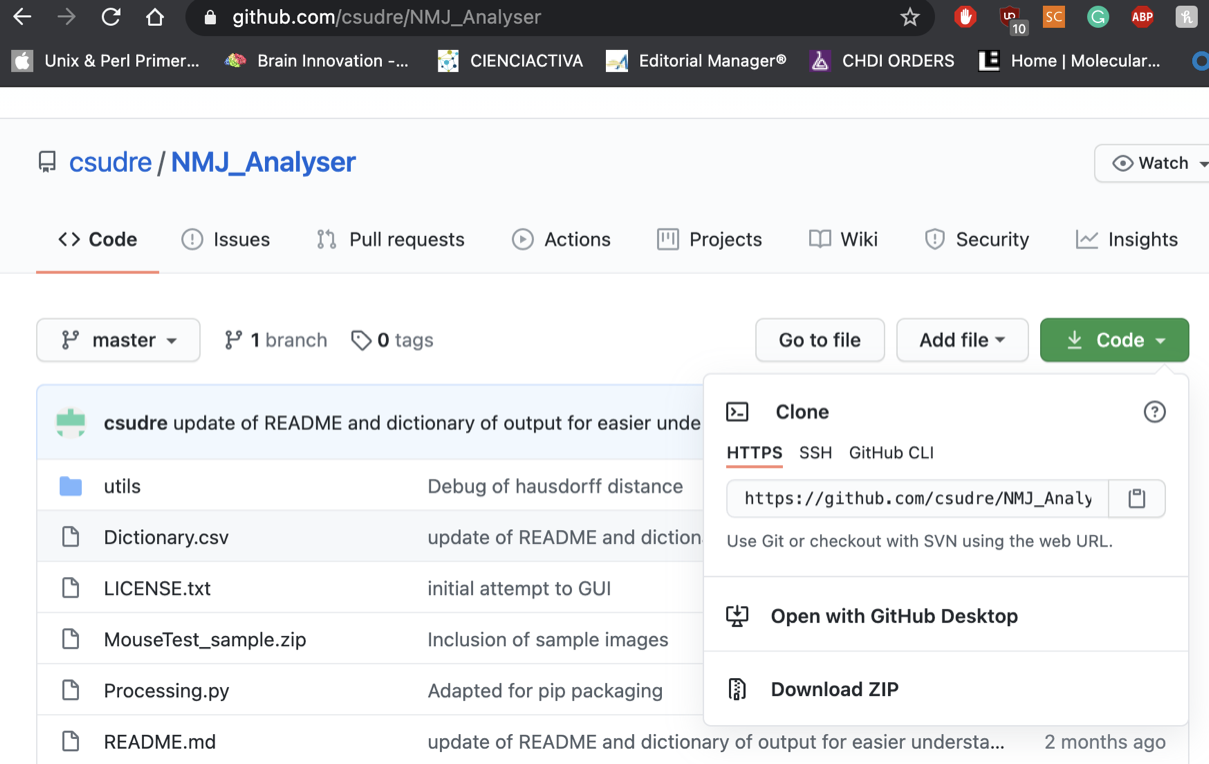


- Identified and unzip the MouseTest_sample.zip. file. You will find three files: Readme.txt, an .lsm file and a folder named “Image_sample” that contains samples images.
- In the GUI interface window type the “X ” and “Z” values of your images. “X” refers to the pixel size and is dependent of the resolution of the image (“⊃” and “⊃ϒ” axes). ẑ refers to slide interval of the Z-stack (projection). These parameters may vary across labs.
- Threshold is setup at 64. Users can find their own threshold depending of the intensity of their image signals and quality of staining.
- Set up the “path to images” and “path to save output” (see below).

**Mac OSX**


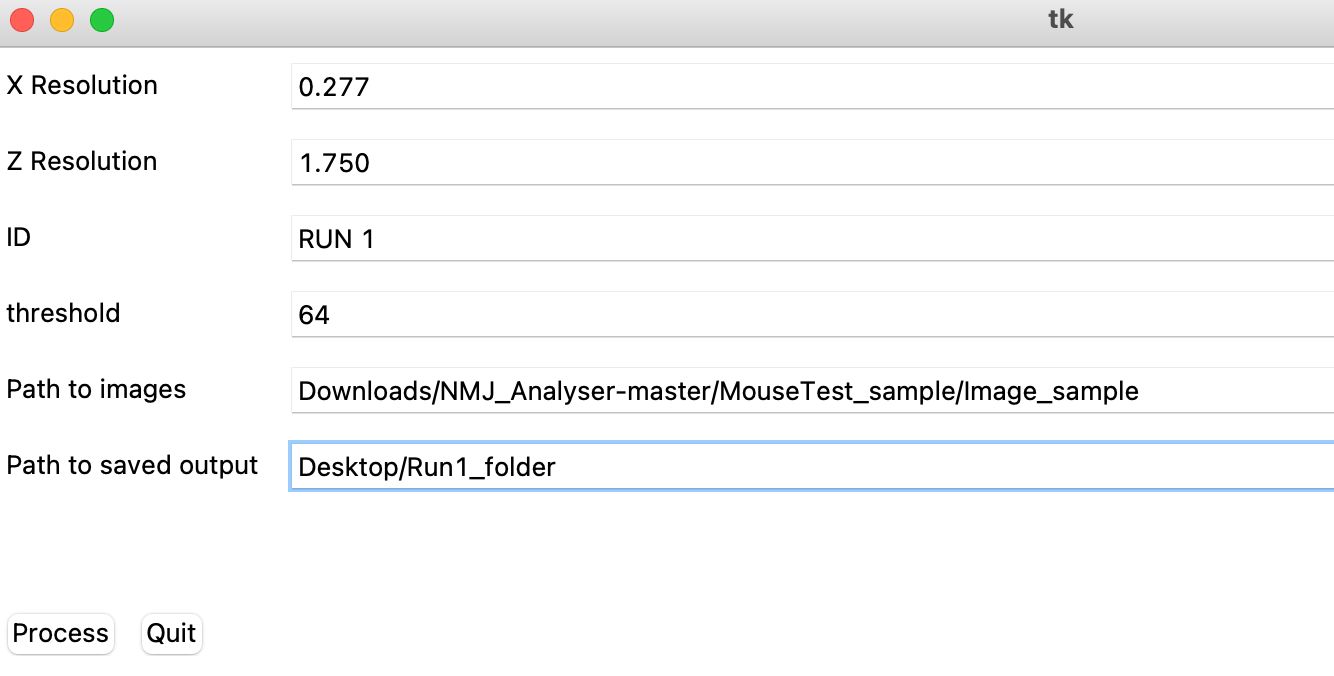


**Windows**


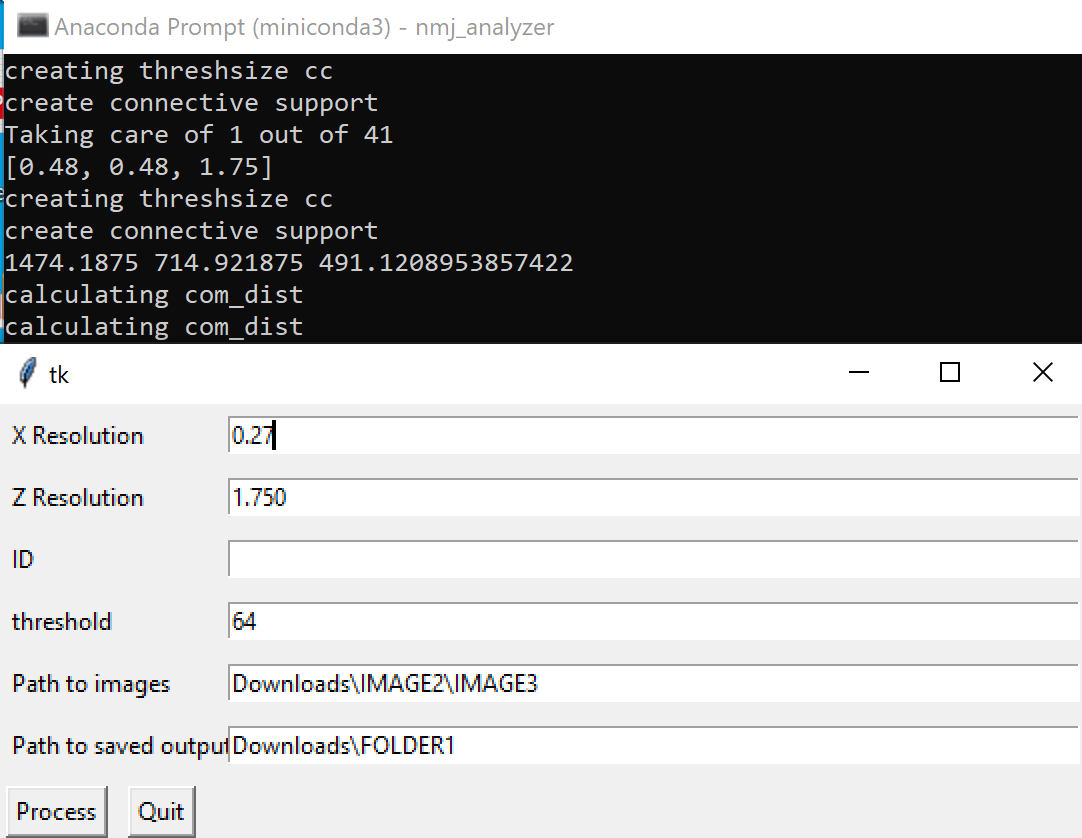


- To run NMJ-Analyser, click on “Process”. Depending on the machine, it may take few seconds to minutes.
- This generates the “ExtractedTableFinFilled_.csv” file, and other three files, on the output directory (here Desktop), which contains the raw data for the sample image. Full description of the variables output can be found in the dictionary.csv at https://github.com/csudre/NMJ_Analyser.

**Working with your own images**

- 3D stacks should be converted into individual nerve terminal and endplate files (plane view, .TIFF,.JPEG or .PNG). See the image_sample folder downloaded from <https://github.com/csudre/NMJ_Analyser>
- Threshold of images is an important part when using NMJ-Analyser. Users may find the suitable threshold by testing if our software can detect all NMJs with full parameters in a given figure. If this happens, stick with your own threshold value and use it for all your analysis.
- Create a folder “My_IMAGES” with images file containing the keyword red or RED (endplate staining), or green or GREEN (nerve staining). The images should be ordered numerically (*i.e.* Mouse1_GREEN_0001.jpg.... Mouse1_GREEN_0010.jpg).

Identify the directory of the input and output folder.

For NMJ-Analyser related topics or NMJ analysis/plotting scripts using R/RStudio, please go here <https://github.com/alanmejiamaza>

**Normalization**

Batch effects are non-biological variation between experiments performed at different timepoints [48,49]. Variables contributing to batch effect (for example, fixation, penetration of antibodies, image thresholding and background staining) impact on the quality and reliability of the immunofluorescence staining. NMJ-Analyser considers the variability between batches by using the same thresholding protocol for all sections and using the voxel size as an input to calculate the 3D NMJ features. We considered a minimum and maximum cut-off size of NMJ structures that are automatically included in the pipeline (Additional File 1: Fig.S1). In cases where multiple batches were compared (i.e. for C57BL/6J and C57BL/6J-SJL male mice), the following normalization protocol was applied: 1) use the same thresholding value and background correction across batches, 2) compare the mean fluorescence intensity (MFI) of nerve terminal and endplate in WT samples, 3) use the cumulative distribution function (ECDF) and Kolmogorov-Smirnov test as a guide of MFI differences across samples (if not normally distributed), and 4) divide the value of each pre- and post-synaptic morphological variable by their MFI. Please notice that when contrast of the staining is strong, the biophysical measures (non-compactness, shape factor, etc) of NMJs may be similar across different thresholds and MFI may not be necessary. Details of the normalization protocol and R/RStudio scripts can be found at Additional File 1: Fig.S1.

Normalization is required when multiples batches are analysed together. Normalization procedure assumes the imaging setup was maintained constant, except for the pixel size.

**Step 1**

- Maintain the same thresholding value and background correction across multiple batches

**Step 2**

- Only in wildtype samples, identify whether the MFI values of nerve terminal or endplate of each batch follows normal distribution (Shapiro-Wilk test, a,b).
- If MFI values do not follow normal distribution, compare them using the cumulative distribution function.
- Compare mean fluorescence intensity (MFI) of nerve terminal and endplate of in WT samples of each batch. Use the cumulative distribution function (ECDF) and Kolmogorov Smirnov (two batches) test or Kruskal-Wallis test (more than two batches) as a guide of MFI differences across samples (Fig.


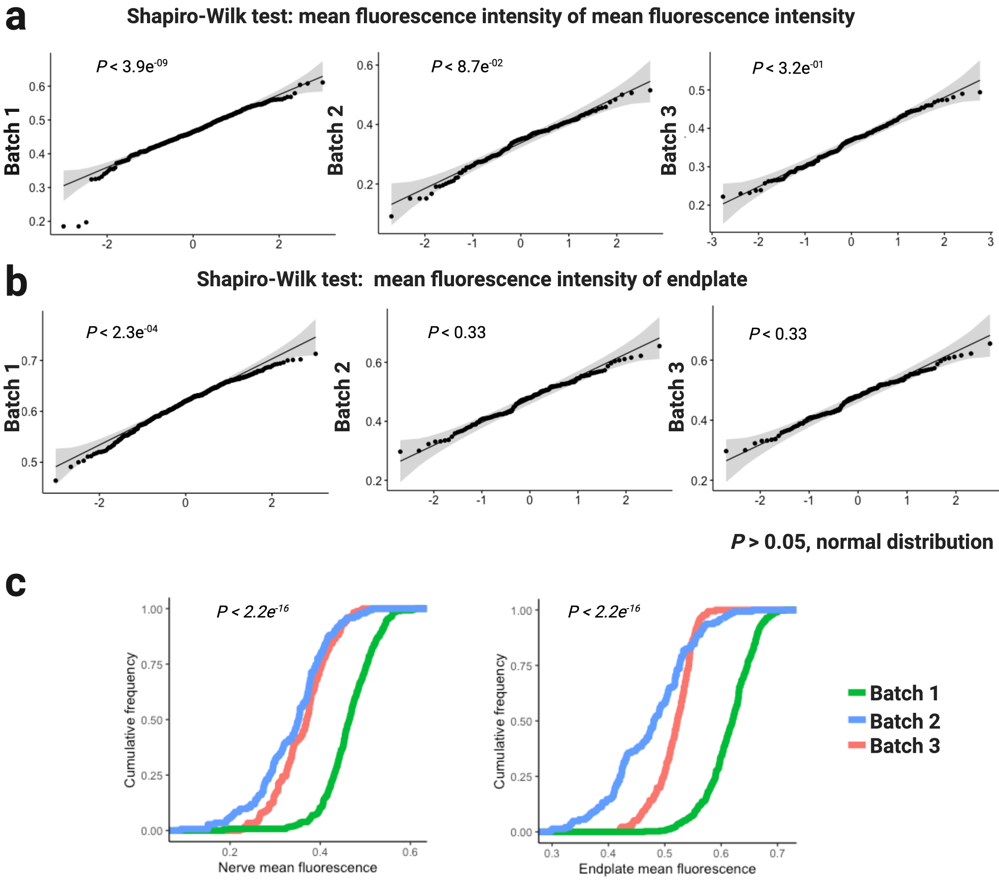


**Fig.S8**

**Step 3**

- Divide the value of each pre- and post-synaptic morphological variable by their corresponding MFI.

An example of normalization process with R/RStudio code for analysis can be found at <https://github.com/alanmejiamaza>

**Manual curation**

Images containing multiple NMJs require manual inspection. This procedure is required to fully identify individual NMJs and avoid poor counting. Thus, it is possible that two or more NMJs can be counted as one if they are close (<20μm).

**Step1**

- Download and install ITK-SNAP viewer: <http://www.itksnap.org/pmwiki/pmwiki.php>
- Open the confocal image (.lsm5 format) on a ITK-SNAP.
- Identify each NMJ obtained from NMJ-Analyser .csv output by typing the position (x,y,z) on ‘cursor position’ (Fig.S2).

**
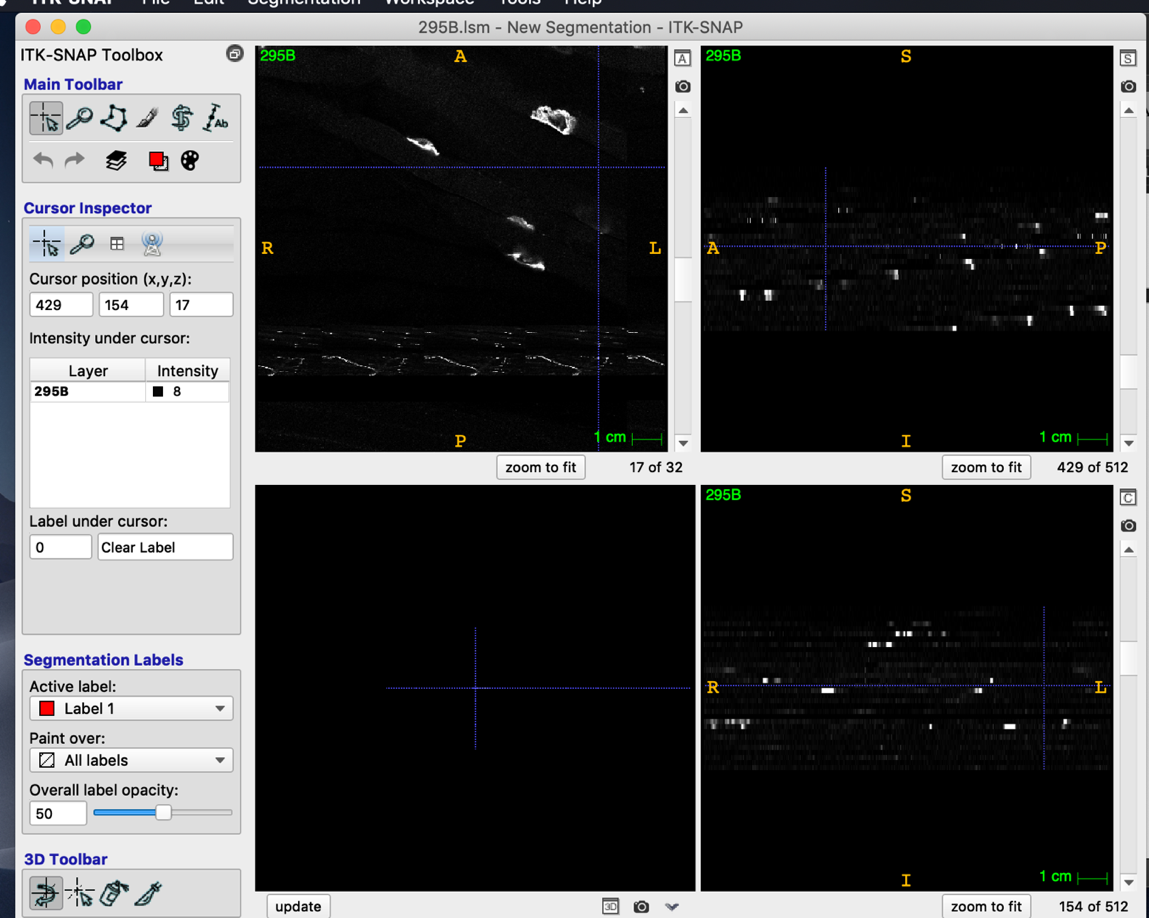
**

**Fig.S9**

**Machine Learning**

Machine learning pipeline and instructions to run the machine learning platform can be downloaded from <https://github.com/SethMagnusJarvis/NMJMachineLearning>

**NMJ-Analyser validation**

We compared the nerve terminal and endplate volume outputs obtained by of NMJ-Analyser and Volocity. The figure below shows strong correlation between outputs obtained by NMJ-Analyser and Volocity (Fig.S3)

**
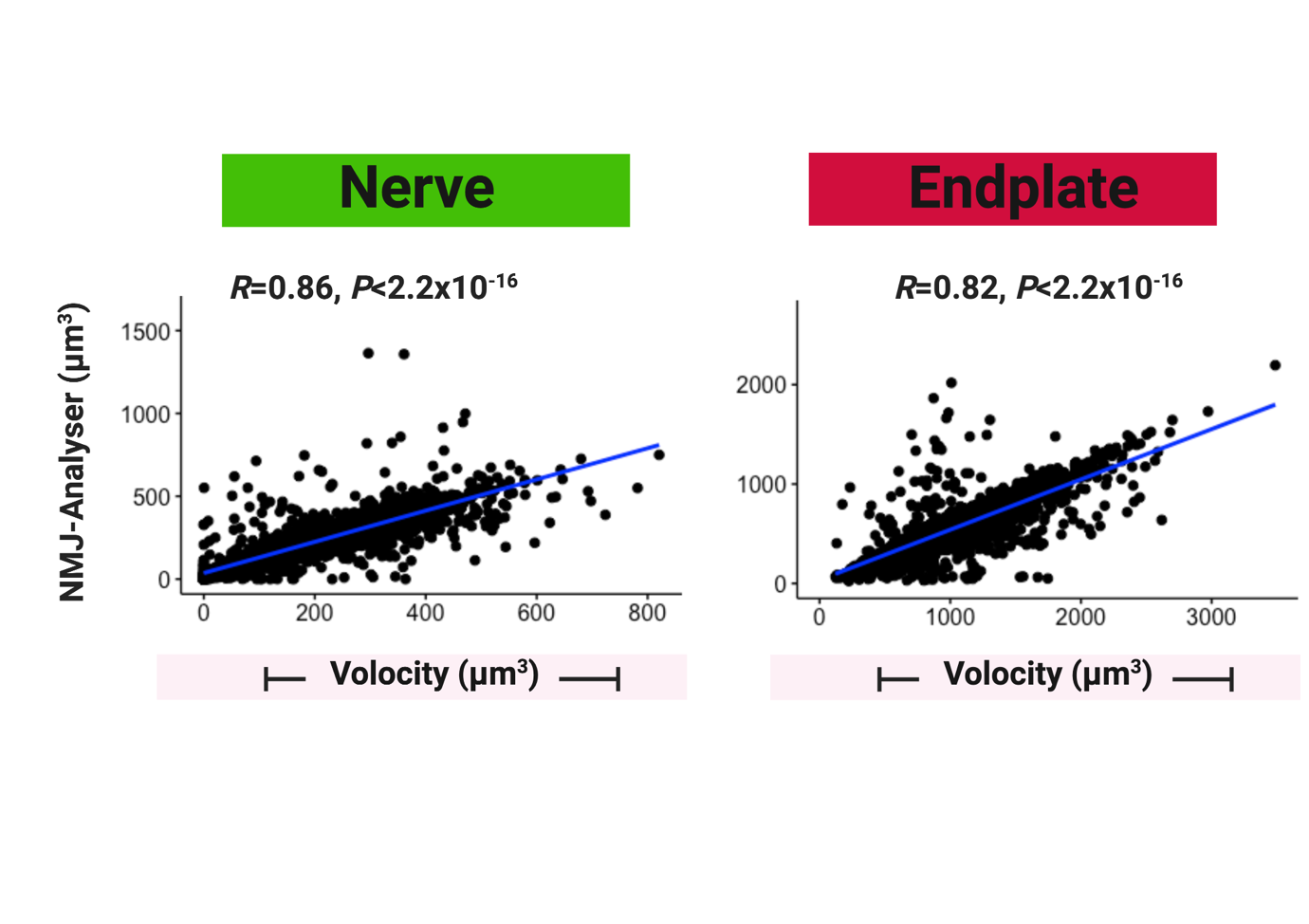
**

**Fig.S10**
